# Genetic discrimination in insurance and employment based on personalized risk stratification for breast cancer screening

**DOI:** 10.3389/fgene.2025.1481863

**Published:** 2025-03-05

**Authors:** Manuela Reveiz, Sarah Bouhouita-Guermech, Kristina M. Blackmore, Jocelyne Chiquette, Éric Demers, Michel Dorval, Laurence Lambert-Côté, Hermann Nabi, Nora Pashayan, Penny Soucy, Annie Turgeon, Meghan J. Walker, Bartha M. Knoppers, Anna M. Chiarelli, Jacques Simard, Yann Joly

**Affiliations:** ^1^ Centre of Genomics and Policy (CGP), McGill University, Montreal, QC, Canada; ^2^ Ontario Health (Cancer Care Ontario), Toronto, ON, Canada; ^3^ CHU de Québec-Université Laval Research Center, Quebec City, QC, Canada; ^4^ CHU de Québec-Université Laval, Quebec City, QC, Canada; ^5^ Département de Médecine Familiale et de Médecine D’urgence, Université Laval, Quebec City, QC, Canada; ^6^ Faculty of Pharmacy, Université Laval, Quebec City, QC, Canada; ^7^ CISSS de Chaudière-Appalaches Research Center, Lévis, QC, Canada; ^8^ Department of Social and Preventative Medicine, Faculty of Medicine, Université Laval, Quebec City, QC, Canada; ^9^ Université Laval Cancer Research Center, Quebec City, QC, Canada; ^10^ Department of Public Health and Primary Care, University of Cambridge, Cambridge, United Kingdom; ^11^ Dalla Lana School of Public Health, University of Toronto, Toronto, ON, Canada; ^12^ Department of Molecular Medicine, Faculty of Medicine, Université Laval, Quebec City, QC, Canada

**Keywords:** breast cancer, risk-stratified breast cancer screening, polygenic risk score, genetic discrimination, canadian insurance legislation, canadian employment legislation

## Abstract

**Background:**

The Breast and Ovarian Analysis of Disease Incidence and Carrier Estimation Algorithm (BOADICEA) incorporates the effects of common genetic variants, from polygenic risk scores, pathogenic variants in major breast cancer (BC) susceptibility genes, lifestyle/hormonal risk factors, mammographic density, and cancer family history to predict risk levels of developing breast and ovarian cancer. While offering multifactorial risk assessment to the population could be a promising avenue for early detection of BC, obstacles to its implementation including fear of genetic discrimination (GD), could prevent individuals from undergoing screening.

**Methods:**

The aim of our study was two-fold: determine the extent of legal protection in Canada available to protect information generated by risk prediction models such as the BOADICEA algorithm through a literature review, and then, assess individuals’ knowledge of and concerns about GD in this context by collecting data through surveys.

**Results:**

Our legal analysis highlighted that while Canadian employment and privacy laws provide a good level of protection against GD, it remains uncertain whether the Genetic Non-Discrimination Act (GNDA) would provide protection for BC risk levels generated by a risk prediction model. The survey results of 3,055 participants who consented to risk assessment in the PERSPECTIVE I&I project showed divergent perspectives of how the law would protect BC risk level in the context of employment and that a high number of participants did not feel that their risk level was protected from access and use by life insurers. Indeed, 49,1% of participants reckon that the level of breast cancer risk could have an impact on a woman’s ability to buy insurance and 58,9% of participants reckon that a woman’s insurance might be cancelled if important health information (including level of breast cancer risk) is not given when buying or renewing life or health insurance.

**Conclusion:**

The results indicate that much work needs to be done to improve and clarify the extent of protection against GD in Canada and to inform the population of how the legal framework applies to risk levels generated by risk prediction models.

## 1 Introduction

The occurrence of breast cancer (BC) in Canada is expected to continue to rise in the next 2 decades underscoring the necessity of improving prevention and screening practices (Poirier et al., 2019). Evidence demonstrates that breast screening with mammography has contributed to decreasing BC mortality (Zielonke et al., 2020; Klarenbach et al., 2018; Nelson et al., 2016). Emerging evidence suggests that personalized risk assessment can improve screening outcomes and risk reduction interventions by targeting those individuals most likely to benefit and could lead to improvement in survival and in quality of life as well as to more efficient allocation of healthcare resources (Pashayan et al., 2020; McWilliams et al., 2022). The risk of BC varies substantially among individuals, with a large proportion of cases occurring in a minority of people who are most susceptible to developing the disease (Pharoah et al., 2008). Individuals at increased risk can be identified through a combination of genetic and lifestyle/hormonal risk factors. Currently known genetic factors include common low penetrance genetic variants, which can be combined as polygenic risk score (PRS) (Mavaddat et al., 2019; Yang et al., 2023) and rarer gene variants that confer higher risks (Breast Cancer Association Consortium, 2021). The latter include deleterious variants in *BRCA1, BRCA2, PALB2* and *TP53*, which confer a high risk of the disease, and variants in *ATM, CHEK2, BARD1, RAD51C* and *RAD51D*, which confer more moderate (∼2-fold) risks.

The Breast and Ovarian Analysis of Disease Incidence and Carrier Estimation Algorithm (BOADICEA) incorporates the effects of common genetic variants, summarized in a PRS, the effects of pathogenic variants in major BC susceptibility genes, lifestyle/hormonal risk factors, mammographic density, and cancer family history to predict the risk levels of developing breast and ovarian cancer (Lee et al., 2019). The PERSPECTIVE I&I (Personalized Risk Assessment for Prevention and Early Detection of Breast Cancer: Integration and Implementation) project, is a translational endeavor that uses BOADICEA to provide cost-effective risk-based screening and interventions as well as risk stratification in 3 levels (average, higher than average, and high risk levels) to identify best practices for implementation within the context of the universal healthcare coverage provided by Canada’s healthcare programs (Brooks et al., 2021). Its application is already integrated in the CanRisk web-tool which assists healthcare professionals in BC risk calculations (Brooks et al., 2021).

However, if accessed and used by insurers and employers, risk assessment can become a source of negative discriminatory treatment similar to what is known as genetic discrimination (GD) (Nature medicine 2021; Yanes et al., 2024). GD occurs when individuals or groups are negatively treated because of their actual genetic characteristics (Otlowski et al., 2012). Several studies have reported that one of the main concerns individuals often raise regarding genetic testing is the risk to become ineligible for insurance and employment (Lapham et al., 1996; Lewis and Green, 2021; Yanes et al., 2019). Such concerns led to the adoption of the Genetic Non-Discrimination Act (2017) by the Canadian government in order to protect at-risk individuals (Joly et al., 2021). However, the limited scope of the GNDA and its specific formulation may limit its effectiveness in addressing some incidents of GD (Young and Thrasher, 2020). One such potential gap is that the outcome of risk prediction models, including the BOADICEA algorithm, may not be covered under the GNDA, because strictly speaking they are not genetic test results.

Given this possibility, it was one of the objectives of the PERSPECTIVE I&I project to determine the extent of legal protection available to protect BC risk level generated by risk prediction models such as the BOADICEA algorithm and to determine individuals’ knowledge of, and concerns about GD. The first part of our manuscript presents a legal review of existing legal protections against GD in Canada and comments on their applicability to risk information generated by risk assessment tools. The second part presents the results from a large-scale prospective cohort study of Canadian women concerning their perception of GD risk associated with undergoing BC risk level assessment with a risk stratified approach.

## 2 Materials and methods

In the first part of this paper, our legal analysis drew upon the current policy literature, to identify the Canadian legal framework applicable to outcomes generated by risk prediction models, including the BOADICEA model. This legal review sought to clarify whether the outcome of risk prediction models and algorithms that account for genetic tests results benefit from the same protection against GD as genetic test results in Canada. The second part of this paper aimed to report the current knowledge and perceptions of Canadian women who underwent BC risk assessment regarding the possible use of their BC risk generated by risk prediction algorithms by Canadian insurers and employers, drawing on responses from a questionnaire. This study is part of the broader PERSPECTIVE I&I project in which Walker et al.‘s paper describes in detail the methodology (Brooks et al., 2021; Walker et al., 2024). This study has been approved by the Ethics Research Committees of the CHU de Québec-Université Laval (MP-20-2020-4670), McGill University (A12-B65-18A), University of Toronto (00036881), Grand River Hospital (2020-0709), McMaster University (11468), St. Michael’s Hospital (19-220), Sunnybrook Health Sciences Centre (2255), University Health Network (19-5340) and Queens University (6030732 EPID-712-20). Informed consent was obtained from all subjects involved in the study.

### 2.1 Legal analysis

For our legal analysis, we gathered data from Canadian laws, regulations and court cases as well as scholarly literature on GD in insurance and employment. This review focused on two main areas: 1) the question of statutory interpretation (interpretation of laws and regulation), and 2) laws and regulations applicable to GD in Canada. Our goal was to assess the susceptibility of risk prediction models to GD, setting the foundation for our empirical study. We sourced information from various search engines, including Web of science, Lexis Advance Quicklaw, Westlaw Canada and Google scholar. The inclusion criteria focused on literature discussing the legal interpretation or construction of statutes in Canada, as well as literature on GD in Canada, relevant laws, policies, regulations or the GNDA since 2005.

### 2.2 Development of the survey questionnaire

Throughout the PERSPECTIVE I&I project, 3 questionnaires were used at different times: first, a pilot questionnaire (at study entry), then a follow-up questionnaire (at the time of risk level communication) and finally, the 1-year follow-up questionnaire (about 1 year after risk level communication). In this paper, we focus on the follow-up questionnaire at the time of risk level communication. Our questionnaire (Alarie et al., 2021) was designed in English and French by a team of experts from the PERSPECTIVE I&I project, pilot tested and revised based on the results. The questionnaires were administered, between March 2020 and October 2022, in both paper and online format in Ontario but only in online format in Quebec. The participants in this analysis are women, who were recruited from Ontario and Quebec, who had undergone BC risk assessment and had recently received their BC risk level, and who have completed the follow-up questionnaire (at the time of risk communication). Participants were not tested for rare variants in susceptibility genes. The questionnaire collected information on screening and diagnostic breast imaging or procedures, attitudes towards BC, mammography and BC risk information, the use of BC risk information and general health. This paper will focus on questions relating to the use of an individual’s BC risk information which were developed by GD experts who are part of the Genetic Discrimination Observatory (GDO, 2024). The selected statements focus on participants’ perceptions regarding whether information about their risk of BC could be used by others (e.g., insurance companies, employers) to discriminate against them. An entry questionnaire administered at study enrollment collected participant sociodemographic data which are also reported here, and the methods used were previously published (Walker et al., 2024).

#### 2.2.1 Data analysis of the questionnaire survey

Descriptive statistics were used to present participant sociodemographic and health characteristics. To assess the association between predictor variables and primary outcomes, we conducted bivariate analyses. Variables used in bivariate analysis included age at risk assessment (years); nativity; visible minority group membership; BC risk level; family history of breast cancer; life or health insurance status; marital status; highest level of education; employment status; and province of residence. Certain predictors, such as education, family history, and employment status, were considered of inherent importance and were included in the analysis, irrespective of bivariate findings. For all other predictor variables, only those with a significance level (p-value) of less than 0.10 in the bivariate analyses and not highly correlated with other variables (correlation matrix threshold of >0.8) were considered for inclusion as predictors in subsequent multivariate models. The five questions used, and their respective outcomes are described in Table 1.

**TABLE 1:** Questions to participants, alongside the correct answer, to assess their knowledge or concern on the issue as well as their perception of the legal protections that are available.

Questions	Answer	Primary outcome
1. *In some circumstances, a woman applying for a job who does not give permission to access her medical record might lose the job opportunity*	*False*	*Presents knowledge on issue*
2. *The level of breast cancer risk has no impact on a woman’s ability to get a job*	*True*	*Presents knowledge on issue*
3. *Employers may request access to a woman’s medical record when she is applying for a job (including her level of breast cancer risk), to assess specific health aspects related to the job*	*False*	*Presents knowledge on issue*
4. *A woman’s insurance might be cancelled if important health information (including level of breast cancer risk) is not given when buying or renewing life or health insurance*	*NA*	*Perception of legal protection*
5. *The level of breast cancer risk has no impact on a woman’s ability to buy insurance*	*NA*	*Perception of legal protection*

^a^ NA: not available.

Multivariate regression analyses were performed to identify independent factors associated with two primary outcomes across five questions. The models aimed to determine the influence of various factors on participants' knowledge of the law and their perceptions related to it while accounting for potential confounders and effect modifiers. We employed multivariable polytomous logistic regression models to estimate odd ratios for each of the seven questions. The outcomes for question 1–3 were the odds of “presenting knowledge on the issue”. A participant with a correct answer was considered to present Knowledge on the Issue, whereas a participant with an incorrect answer or that did not know the answer was considered to present Low Knowledge on the Issue. The outcomes for question 4–5 were the odds of Feeling Protected by the Law since the information on whether the law provides protection from GD or not, in this context, is not available. If the woman answered TRUE to Question 4, she was considered to Feel Weak Protection by the Law, whereas a woman answering FALSE was considered to Feel Protected by the Law. If the participant answered FALSE to Question 5, she was considered to Feel Weak Protection by the Law, whereas a woman answering TRUE was considered to Feel Protected by the Law. Model assumptions, including linearity, independence of errors, and absence of multicollinearity, were evaluated. The Akaike Information Criterion (AIC) was employed for model selection in our analysis. We also considered other factors such as domain knowledge to inform model selection. Statistical analyses were conducted using SAS software (SAS Institute Inc., Cary, NC), version 9.4.

## 3 Results

### 3.1 Is the outcome of risk prediction models protected by the canadian legal framework preventing GD?

#### 3.1.1 Genetic discrimination: Canadian experience and legal protection

The issue of GD in Canada was first evoked in 1991 by Knoppers in a study completed for the Law Reform Commission of Canada (Knoppers, 1991). Following this early warning, the occurrence of GD in life insurance in Canada was confirmed in the context of Huntington’s disease by Bombard et al., in 2008 (Bombard et al., 2008) and to a lesser extent in the context of other highly inheritable genetic diseases such as BC (Dalpé et al., 2017) and sudden arrhythmia death syndromes (Mohammed et al., 2017) by subsequent studies. There were also concerns about GD expressed in the context of employment (Otlowski et al., 2012). Nonetheless, GD in employment was generally thought to be less of a risk in Canada than in the United States due to significant differences in the employment and healthcare structure between the two countries, notably the existence of a universal healthcare system in Canada (Pullman and Lemmens, 2010).

According to Canadian insurance law, which is relatively uniform across the country, insurance applicants should disclose any information (including health information) that could influence an insurer in pricing an insurance contract, assessing the risk to be insured, or deciding to accept an application (Lemmens, 2003). To facilitate the realization of this duty, application forms for life insurance contracts will usually include a health questionnaire regarding the person to be insured that needs to be truthfully completed.

In contrast, Canadian employers can ask specific health questions or impose a medical exam on future employees only if they directly relate to their capacity to carry out the job they are applying for. Furthermore, in some Canadian provinces (e.g., Ontario), such investigation is only permissible once an employment offer has been made to a prospective candidate. Employers are, however, not permitted to make broad inquiries into the health of their prospective employees or to ask them to provide access to their entire medical record (Supreme Court of Canada, 1998b; 2000). Thus, even before the adoption of a specific GD prohibition, the legal protection against GD in Canada was relatively robust in the context of employment. Additionally, given the nature of the Canadian healthcare system, basic health insurance was not an issue. However, the degree of protection provided was however deemed insufficient in the context of life insurance.

In addition to this generic protection applying to all health data, following a longstanding effort led by the Huntington Society of Canada, and eventually, the Canadian Coalition for Genetic Fairness, the GNDA was finally adopted in 2017 (Genetic Non-Discrimination Act, 2017). Nonetheless, a constitutional challenge led to a prolonged period of uncertainty regarding the validity of this law until its constitutionality was finally confirmed by the Supreme Court of Canada in 2020 (Supreme Court of Canada, 2020). Key provisions of the GNDA are to the effect that parties providing goods or services to, or entering into a contract with, a person cannot require that person to take a genetic test or to disclose their genetic results. Such parties are also forbidden to collect, use, or disclose a person’s genetic test results, no matter how those results were obtained (Section 3). Importantly, a genetic test is defined in the GNDA (Section 2) as ‘‘a test that analyzes DNA, RNA or chromosomes for purposes such as the prediction of disease or vertical transmission risks, or monitoring, diagnosis or prognosis.’’ A first study by Fernando et al. (2024) of the impact of the GNDA on practices of Canadian life insurance companies found the GNDA only had a minimal impact (Fernando et al., 2024).

#### 3.1.2 Information obtained from risk prediction models: Falling through the cracks of the GNDA?

Risk prediction algorithms use predictors (covariates) to estimate the probability or absolute risk that a given outcome is present or will occur within a specific time span in a person with a particular predictor profile. Predictors are used by risk prediction models to assess the risk range from patients’ characteristics (e.g., age and sex), history and physical examination results, imaging, electrophysiology, blood, urine, coronary plaque, and, genetic markers (Moons et al., 2012). Such models can assess how a patient might respond to treatment, or whether an individual is likely to develop cancer in the first place. Given scientific validity and clinical utility, information generated by these algorithms can be used by physicians and public health authorities to make more coherent and informed therapeutic or preventive decisions (Moreno et al., 2023).

As an illustration, the BOADICEA risk prediction algorithm considered in the context of the PERSPECTIVE I&I project predicts breast and ovarian cancer risk based on both genetic and nongenetic factors. The algorithm takes into account the effects of common genetic variants, summarized in a PRS, in addition to the effects of pathogenic variants in major breast cancer susceptibility genes which were not considered in this study, other lifestyle/hormonal predictors, mammographic density, and cancer family history. The outcome of BOADICEA’s assessment takes the form of a lifetime breast cancer risk, or age-specific shorter time interval, such as 5-year, 10-year breast cancer risk for a woman. Thereafter, this information can be used for risk stratification relative to the rest of the population. For example, in the context of the PERSPECTIVE I&I project such a risk level is expressed as 1) average risk, 2) higher than average risk or 3) high risk (Pashayan et al., 2021). A screening action plan was proposed for each risk level as part of the PERSPECTIVE I&I study. It is hoped that this information will be used by clinicians and public health services to provide more personalized and appropriate breast cancer preventive care to individuals (Pashayan et al., 2020; Brooks et al., 2021).

But, once ready for clinical use, *could the risk level derived from the multifactorial risk assessment outcome of the BOADICEA algorithm be used by insurers or employers to discriminate against individuals, or would this be prevented by the GNDA?* In the preceding section (Section 3.1.1), we explained that the GNDA as it is formulated would prevent both employers and insurers from seeking the result of a genetic test (as defined in the Act) in the context of an agreement. The question that is raised regarding the protection of ‘‘risk level’’ then becomes one of statutory interpretation and the need to consider the meaning of “genetic results” and “genetic tests” under the GNDA. Where Canadian courts once applied the principle of literal interpretation to solely focus on the text of the law in matters of interpretation, more recently, there has been a trend to consider the broader context and objective of the law (Supreme Court of Canada, 1972). As held by the Supreme Court in *Rizzo and Rizzo shoes* (Supreme Court of Canada, 1998b), the current principle now warrants that “the words of an Act are to be read in their entire context and in their grammatical and ordinary sense harmoniously with the scheme of the Act, the object of the Act, and the intention of [the legislature]’’ (Supreme Court of Canada, 1998a). Yet, even when applying this more permissive method, if the words used are precise and unequivocal, the ordinary meaning of these words play a dominant role in the interpretive process (Supreme Court of Canada, 2005).

The intent of the legislator here is, arguably, to prevent GD in Canada. To achieve this, the GNDA prohibits that the results of genetic tests be communicated in the context of contracts of goods and services (Government of Canada, 2017). The legislator also clearly defined what should be considered a genetic test in the second article of the GNDA as a: “test that analyzes DNA, RNA or chromosomes for purposes such as the prediction of disease or vertical transmission risks, or monitoring, diagnosis or prognosis” (Government of Canada, 2017).

Applying the law which comport a clear definition, to the current case, would mean that the GNDA should apply only to genetic test involving the use of DNA, RNA or chromosomes. A broader interpretation could lead to the inclusion of a large number of medical test and diagnostics that are based, in part, on genetic information, and tests based on other ‘OMICS’ results. For example, family history of hereditary diseases, which life insurers are allowed to collect through their underwriting questionnaire (Mannette, 2021). Arguably, risk levels that rely on a variety of data sources, are different from genetic results. To put it simply, while the results of a genetic test would tell us about the genetic profile of a person, including DNA and RNA mutations that could be associated with genetic diseases susceptibility, the outcome of a risk prediction algorithm is a lifetime or age-specific risk assessment which can be used for risk stratification into a risk category compared to a population mean. This leads us to conclude that ‘‘risk levels’’ may not be protected by the GNDA.

Given our finding that the GNDA as currently formulated, may not protect predicted BC risk information, we need to look back at the protection provided by other Canadian laws (ex. on data privacy, health research, bioethics, human rights, etc.) to see if it is of help in this situation. Reviewing such legislation (see preceding Section 3.1.1) (Ontario Human Rights Code, 2015; Canadian Human Rights Act, 1985; Canada Labour Code, 1985; Genetic Non-Discrimination Act, 2017) leads us to the conclusion that nothing in this legal framework would prevent a life insurer from requesting access to the risk level produced by the BOADICEA algorithm (or any other risk prediction model), if the risk level is of a nature that would influence a reasonable insurer in pricing an insurance, assessing the risk or, deciding to accept an application. Of course, such information could only be obtained by the insurer with the consent or voluntary disclosure of the applicant. But such consent to verify health information is usually provided to the insurer through a standard clause contained in most life insurance application forms in Canada (Lemmens, 2003). In the context of employment, the risk appears much lower that this information could be misused. Indeed, an employer can only request information that directly relates to a prospective employee’s capacity to carry out the job he/she is applying for (Supreme Court of Canada, 1998b; 2000). Referring to our example, clearly the outcome of the BOADICEA model would not fit that requirement. This assessment of protection available for risk information produced by risk prediction models, and more specifically of the outcomes of the BOADICEA algorithm, leads us to the unsettling conclusion that it is uncertain that this information is protected by the GNDA and that it could, therefore, be requested by life insurers in the context of an application to purchase a new life insurance policy or an additional amount of health insurance.

### 3.2 Knowledge and perceptions of Canadian individuals regarding the possibility that risk scores be used by Canadian insurers or employers

**FIGURE 1:**
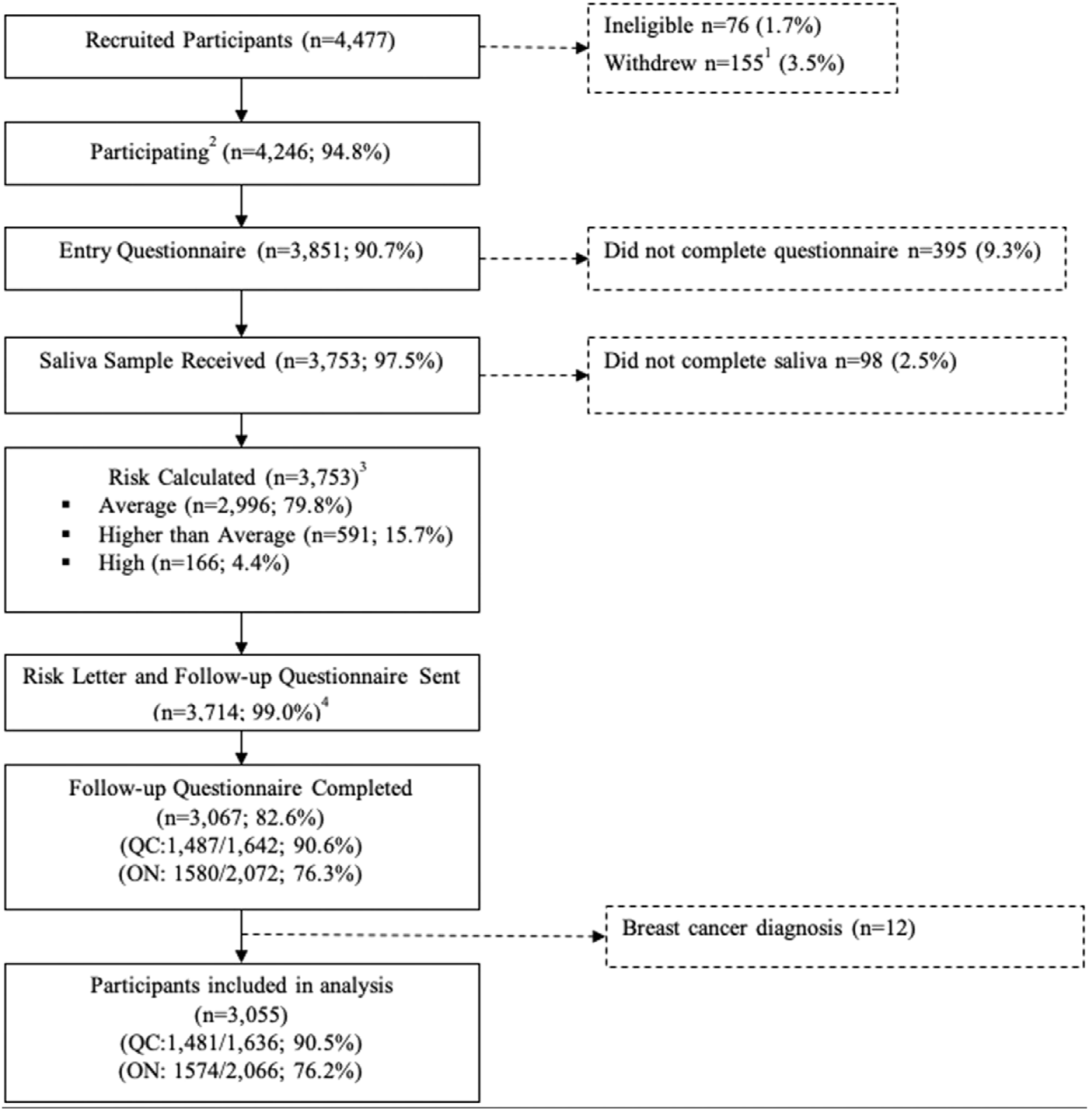
Recruitment and data collection of Quebec and Ontario PERSPCETIVE I&I participants aged 40–69 years. 1. Includes n = 150 who withdrew before risk assessment with data conservation and n = 5 who withdrew without data conservation. 2. Includes n = 21 who withdrew with data conservation after risk assessment. 3. Risk estimated without PRS for n = 3. 4. N = 39 Ontario participants did not consent to know their risk level.

Questions raised by genetics, risk prediction models and legal protection against discrimination are complex and the answers we found through our legal analysis represent the most likely outcome given the most recent interpretation of the Canadian law and court precedents. Yet, they constitute a most probable scenario rather than a certainty regarding the application of the law in such circumstances. Hence, the interest in determining how much Canadian individuals know about this topic and whether the risk of discrimination is a concern for individuals considering providing their information for risk assessment or who have done so in the recent past. A total of 4477 women were recruited to participate in the PERSPECTIVE I&I pre-implementation prospective cohort study. A total of 3,714 women were invited to complete a follow-up questionnaire at the time of risk communication, in which 3,067 completed, giving a response rate of 82.6%. A final sample of 3,055 participants were included for the current analysis (Figure 1).

A total of 1,574 women were from Ontario and 1,481 were from Quebec. The mean age of participants in the study was 58 years (61 years in Ontario and 55 in Quebec). The majority had pursued education past high school, and 85.5% had life and/or personal health insurance at the time of survey participation. The sociodemographic and health characteristics of these participants are presented in Table 2.

**TABLE 2:** Sociodemographic and health characteristics of PERSPECTIVE I&I participants, from Ontario and Quebec, (n = 3,055).

	Results per province n (%)		
	Ontario n = 1,574	Quebec n = 1,481	Total n = 3,055 (100%)
Age			
40–49	7 (0.4%)	442 (29.8%)	449 (14.7%)
50–59	651 (41.4%)	583 (39.4%)	1,234 (40.4%)
60–70	916 (58.2%)	456 (30.8%)	1,372 (45.0%)
*Missing*	—	—	—
Nativity			
Canada	1,239 (78.7%)	1,419 (95.8%)	2,658 (87.0%)
Other	325 (20.7%)	59 (4.0%)	384 (12.6%)
*Missing*	10 (0.6%)	3 (0.2%)	13 (0.4%)
Visible minority			
Visible minority	156 (10.0%)	21 (1.4%)	177 (5.8%)
Not a visible minority	1,404 (89.2%)	1,418 (95.8%)	2,822 (91.4%)
Don’t know/prefer not to answer/*missing*	14 (0.9%)	42 (2.8%)	56 (1.8%)
Risk level			
Average	1,292 (82.1%)	1,094 (73.9%)	2,386 (78.1%)
Higher than average	243 (15.4%)	276 (18.6%)	519 (17.0%)
High	39 (2.5%)	111 (7.5%)	150 (4.9%)
*Missing*	—	—	—
Family History of breast cancer^5^			
Family history of breast cancer	653 (41.5%)	866 (58.5%)	1,519 (49.7%)
No family history of breast cancer	921 (58.5%)	615 (41.5%)	1,536 (50.3%)
*Missing*	—	—	—
Life or Personal Health Insurance			
Has Insurance	1,121 (71.2%)	1,279 (86.4%)	2,400 (78.6%)
No Insurance	294 (18.7%)	114 (7.7%)	408 (13.3%)
Prefer not to answer/*missing*	159 (10.1%)	88 (5.9%)	247 (8.1%)
Marital status			
Married/common law	1,178 (74.8%)	1,124 (75.9%)	2,302 (75.4%)
Single/widowed/divorced/separated	377 (24.0%)	354 (23.9%)	731 (23.9%)
Prefer not to answer/*missing*	19 (1.2%)	3 (0.2%)	22 (0.7%)
Education			
University Bachelor’s degree or above	834 (53.0%)	746 (50.4%)	1,580 (51.7%)
College/Registered Apprenticeship/trades certificate	508 (32.3%)	576 (38.9%)	1,084 (35.5%)
High school diploma or below	217 (13.8%)	157 (10.6%)	374 (12.2%)
Prefer not to answer/*missing*	15 (1.0%)	2 (0.1%)	17 (0.6%)
Employment status			
Employed	838 (53.2%)	1,007 (68.0%)	1845 (60.4%)
Unemployed	128 (8.1%)	76 (5.1%)	204 (6.7%)
Retired	602 (38.3%)	393 (26.5%)	995 (32.6%)
Prefer not to answer/*missing*	6 (0.4%)	5 (0.3%)	11 (0.4%)

#### 3.2.1 Descriptive results

The sampling population included 3,055 women residing in Ontario or Quebec. The percentages of different levels of knowledge (correct, wrong or doesn’t know the answer) on selected statements regarding Canadian GD laws are presented in Table 3. Due to the novelty and yet uncertain scope of the GNDA (see Section 3.1.2), all knowledge-based questions focus on employer-related legislation. The majority of participants exhibited moderate knowledge.

**TABLE 3:** Percentage of women who had knowledge about genetic discrimination laws.

Item		Participant response		
	Answer	Correct answer	Wrong answer	Doesn’t know
1) In some circumstances, a woman applying for a job who does not give permission to access her medical record might lose the job opportunity. (n = 3,009)	False	41.1% (1,237)	23.0% (693)	35.9% (1,079)
2) The level of breast cancer risk has no impact on a woman’s ability to get a job. (n = 3,013)	True	63.8% (1921)	13.3% (401)	22.9% (691)
3) Employers may request access to a woman’s medical record when she is applying for a job (including her level of breast cancer risk), to assess specific health aspects related to the job. (n = 3,003)	False	58.8% (1765)	14.6% (439)	26.6% (799)

Perception based percentages are presented in Table 4. For life insurance-based prompts, participants exhibited low confidence and preoccupation regarding the ability of the legal system to successfully protect them against GD. For the employer-based prompt, a moderate number of participants expressed confidence that the law would protect them against GD.

**TABLE 4:** Percentage of women who felt protected by genetic discrimination laws.

Item	Participant response		
	Felt protected by law	Did not feel protected by law	Doesn’t know
4) A woman’s insurance might be cancelled if important health information (including level of breast cancer risk) is not given when buying or renewing life or health insurance. (n = 3,007)	7.9% (238)	58.9% (1770)	33.2% (999)
5) The level of breast cancer risk has no impact on a woman’s ability to buy insurance. (n = 3,011)	20.5% (617)	49.1% (1,479)	30.4% (915)

Our finding suggests that, still, some participants did not appear to have sufficient knowledge of legal rules to data protection and access to personal information in the context of employment and demonstrated a lack of familiarity with them. Three questions were asked in connection with this (Questions 1,2,3). The first question (Q1) was more general since it concerned the protection of medical records and did not specifically speak about risk prediction models and risk categories. While employment and privacy legislation in Canada clearly does not provide a general access right to the content of medical records to prospective employers, 23% of participants felt that refusing an employer access to the content of their medical record could be grounds for an employer not to hire them and 35.9% stated they could not answer the question. The majority of participants (58.9%), thus, got the first question on the state of the law wrong, or could not answer it.

The second and third questions (2 and 3) asked participants if they thought employers could ask them to provide their risk levels and would be able to use this information as a basis to not hire them. While a majority of participants (63.8%) felt that their risk level should not impact their capacity to find employment, 13.3% of them still felt that employers had the right to ask for access to this information if it was included in their medical file, while 22.9% expressed having insufficient knowledge in the matter. Thus, while a majority of participants provided the right answer to question 2 and 3, a substantial minority did not or said they did not know the answer (Table 3).

The results for Q4 and Q5 reflects that most of our respondents did not feel protected by the law and believed that risk levels aren’t protected under GNDA, in this context, and should be shared with life insurers during applications.

### 3.3 Bivariate and multivariate regression results

To identify potential associations between poor knowledge of GD laws and perception of GD protection within Canada and socio-demographic characteristics, univariate, bivariate and multivariate polytomous logistic regression analyses were conducted. Supplementary Tables S1–S7 that represent these results are found in the Supplementary Material. The following section illustrates the most prominent results.

After completing a bivariate and multivariate analysis, we attempted to identify possible trends that reflect sociodemographic characteristics. However, our results in the employment-based context were inconclusive and did not follow a clear trend. For example, participants with a family history of BC were more likely to give a wrong answer in Q1 (Supplementary Table S1) but less likely to give a wrong answer in Q2 (Supplementary Table S2). Surprisingly, other results showed that participants in Quebec were less likely to give a wrong answer for Q1 (Supplementary Table S1) but more likely to give a wrong answer for Q2 (Supplementary Table S2).

Our bivariate and multivariate analysis also aimed to identify trends related to sociodemographic characteristics in an insurance-based context. Surprisingly, we observed that unemployed participants and those who were part of a visible minority were more likely to feel protected regarding insurance laws. Another unexpected result showed that participants who did not possess insurance or were not interested in obtaining some were less prone to giving the wrong answer to questions Q1 (Supplementary Table S1) and Q3 (Supplementary Table S3). Further, participants with a high school diploma or less were more likely to give correct answers and to feel confident in both knowledge and perception-based statements.

## 4 Discussion

### 4.1 Employment

#### 4.1.1 General observations amongst participants

Results illustrated how individuals still had limited knowledge of rules of access to personal information in the context of employment and lack of familiarity with those rules (Table 3) which was expected as answering the question correctly would require knowledge of complex legal texts applicable in three different jurisdictions federal and provincial (Ontario and Quebec) and in several legal domains including employment and human rights (privacy, non-discrimination) law. The most likely explanation for the results is that most participants did not know what legal protection (if any) applied to risk prediction information against employment discrimination. Adding to the challenge, many of the laws applicable to these domains and relevant to our situation were adopted (e.g., GNDA) or amended (e.g., Act Respecting the Sharing of Certain Health Information, P-09.0001) recently. The result from our survey suggests a great need for public engagement on the topic of GD and more generally discrimination based on other types of predictive health data, so as to promote a greater knowledge of existing legal protections and best practices. Simple and clear tools for everyone need to be developed. The adoption of the GNDA was a missed opportunity for the Canadian government to undertake such broad engagement and engage in a much-needed social debate on this question.

#### 4.1.2 Observations on significant sociodemographic characteristics

Similarly, to our survey results indicating that participants with a family history of BC were more likely to answer Q2 (Supplementary Table S2) correctly compared to Q1 (Supplementary Table S1), the literature shows conflicting viewpoints on whether individuals with a family history of a disease are well-informed about their rights regarding employment. Individuals with a family history of BC could be more knowledgeable about the outcomes of having BC, which could stem from past experiences. However, some authors claim that those with a family history of BC could have elevated fear of GD due to negative experiences their relatives might have encountered (Wauters and Van Hoyweghen, 2016). These negative experiences might shape their understanding of GD.

An individual might be uncertain about statements concerning BC risk levels, as people could become more apprehensive about health information when it reveals a specific medical condition or a predisposition of a disease. This could decrease their trust levels because they fear that their health information can be misused in such cases. Bell et al. indicated that most patients in their study, which aimed to assess participants’ opinions on sharing information from their medical records for research, were willing to share this information for research, as long as they could control access to sensitive data (Bell et al., 2014). Therefore, some individuals might be warier about health data regarding a disease or even a risk category related to a disease as opposed to general health information.

### 4.2 Insurance

#### 4.2.1 General observations amongst participants

The results suggest that the number of participants in our study who did not feel that their risk level data was protected from insurance companies were higher than those who felt protected (Table 4). Participants who did not feel protected believe that risk prediction information is not GNDA protected information and that it should be communicated to a life insurer when applying for insurance. This finding supported by responses to Q4 and Q5 is particularly interesting as it matches our own conclusion in the first section of the paper and highlights the absence of specific life-insurance legislation in Canada that can successfully prevent discrimination in the context of risk stratification using the BOADICEA risk prediction algorithm. We concluded following our legal analysis (Section 3.1) that should a GD case go to court, the scope of the GNDA *could be* interpreted in a way that excludes risk levels from a multifactorial risk prediction model since they are unlikely to be included within the definition of ‘genetic test’ purported by the act. Should the GNDA be found not to apply to these risk levels, the degree of protection afforded to individuals in this context by human rights, data privacy and insurance legislation at the federal and provincial level could be insufficient to protect against discrimination.

As found in the literature, there seems to be a greater level of concern regarding GD in the context of insurance as opposed to employment (Wauters and Van Hoyweghen, 2016). Given the prevailing context of uncertainty and concerns, if BOADICEA is widely implemented in clinical practice for a risk-based BC screening at the population level in Canada, with risk information communicated to treating physicians, individuals may need to communicate their risk level to insurers to avoid the possibility of having their insurance annulled at the request of the insurer for incomplete disclosure of information relevant to the contract (Civil Code of Quebec, 1991). A traditional fallback strategy is for individuals to purchase their life insurance prior to providing data for a BOADICEA risk assessment. This strategy would work because in the case of whole life insurance policies, once the risk has been accepted by an insurer at a given rate, they no longer are allowed to re-assess it if health circumstances change.

#### 4.2.2 Observations on significant sociodemographic characteristics

Many of our results are not supported by the literature and seem counterintuitive. For instance, our results showed that unemployed participants and those who were part of a visible minority were more likely to feel protected regarding GD in the context of insurance, when the existing literature suggests that uninsured individuals in Canada often hold low-income jobs and are recent immigrants (Bunn et al., 2013; Li et al., 2023). Various reasons, such as language barriers, a lack of trust, income levels, could explain why individuals from specific backgrounds would not purchase personal insurance. Another example was that participants who did not own insurance or were not interested in obtaining it were less prone to giving the wrong answer to questions Q1 and Q3. Therefore, additional research is needed to explore other key reasons for not purchasing insurance, as well as to assess whether the public generally trusts insurance companies with their data, especially with the growing use of health data in all aspects of life (Simeon, 2023). It was also found that participants who were not married (legally or under common law) were less likely to feel protected from GD. Even though marital status should not affect the cost of, or access to, health insurance in Canada, it seems that some participants may hold the false assumption that it does (Averett et al., 2013).

Furthermore, research indicates an increase of breast screenings among insured non-elderly women compared to their uninsured counterparts (Tangka et al., 2020). This suggests that participants who have not sought insurance may be more inclined to delay undergoing breast screenings until they secure insurance, if they expect in doing so. This trend could extend to purchasing insurance prior to undertaking their individual risk assessment using the comprehensive BOADICEA risk prediction model. While the literature highlights several reasons why individuals decide to not undergo breast screening-such as discomfort during screening, low willingness of preventive healthcare, reluctance to receive a diagnosis, unwillingness to share genetic information, and lack of information of breast screening in general, further research is needed to explore the relationship between insurance and participation in breast screening (Ghanouni et al., 2020; Kelley-Jones et al., 2021; Kregting et al., 2020; Mbuya-Bienge et al., 2021; Pagliarin et al., 2021; Azar et al., 2022).

Interestingly, participants with a high school degree or lower were more likely to provide correct answers and feel protected for both knowledge and perception-based statements. This finding is surprising as the literature shows that individuals with higher education levels are more likely to have insurance than those with lower levels of education (Cha and Cohen, 2022).

## 5 Limitations

While this study has been conducted rigorously, using well rodded instruments, some results from our participants’ survey were less conclusive. Inconsistent findings can be explained by several other factors. First, both Ontario and Quebec administered questionnaires virtually. However, Ontario offered alternatives (i.e., phone, paper) which might have helped reach more individuals who were aged 60 and above by making it more accessible (Walker et al., 2024). This shows that future studies should provide the same conditions in each demographic group to eliminate possible limitations. Another possible explanation could be that these participants answered the way they did because they felt that even though employers/insurers do not have a legal right of access, abuses still happen in practice. This may indicate a lack of trust in the protection capacity of the law to effectively prevent discrimination rather than to the ignorance of its existence and content. Further, statements made in the questionnaire are fairly complex and could have been misunderstood by participants which could have then led them to make false assumptions. The limited answer options (true, false, do not know) also make it possible that some participants who did not know the law simply guessed the right answer(s).

## 6 Conclusion

This manuscript intended to determine both the extent of legal protection in Canada of information generated by risk prediction models, such as the BOADICEA algorithm, and the knowledge and concerns of individuals about GD in this context. We concluded that Canadian employment and privacy legislation offer a generally good level of protection against GD. However, we did not come to the same conclusion regarding insurance law. To raise the protection of genetic information, the Canadian parliament adopted the GNDA, in 2017. This law prevents imposing genetic testing as well as requesting results from such tests as a requirement for concluding contracts for goods and services. However, our analysis shows that it is uncertain that the protection of the GNDA against GD would apply to a risk level generated following a risk assessment using a risk prediction model.

The results of our survey of individuals having participated in the PERSPECTIVE I&I project showed that many (58.9%) did not know how the law would protect their risk level in the context of employment. This can be explained by the fact that knowledge of complex scientific and legal notions was necessary to answer this question and because of the lack of an information campaign about discrimination risk relating to the outcome from risk prediction models and prevention measures. Looking at the responses provided to the second part of our survey, it appears that most participants did not feel their risk level was protected from access and use by life insurers. This was a surprising finding, although it did echo our own legal analysis as found in the first part of this manuscript. Their responses to insurance questions may be explained by the fact that GD in insurance has received more (negative) spotlight in the media and thus be better understood by the public than in the context of employment.

The results of both the legal analysis and the survey suggest that much work remains to be done to provide individuals a better understanding of protections from discrimination that are currently available to risk information generated by risk prediction models using both genetic and non-genetics risk factors. This challenge could be addressed in two broad steps: 1) clarifying whether the GNDA applies to risk prediction information that took accounted for genetic data, 2) developing targeted, accessible information campaigns to educate individuals about their rights and protective measures in this context. Of course, the devil is in the details and convincing politicians that discrimination based on predictive health data was not fully resolved by the adoption of the GNDA and that more work is needed both in term of legal reform and concomitant public communication promise to be a challenging undertaking.

## Data Availability

The original contributions presented in the study are included in the article/Supplementary Material, further inquiries can be directed to the corresponding author.
