# Supplementary material for: Genetic discrimination in insurance and employment based on personalized risk stratification for breast cancer screening
Source: Front Genet. 2025 Mar 5;16:1481863. doi: 10.3389/fgene.2025.1481863 (PMC11919849; doi:10.3389/fgene.2025.1481863)
Supplement: Supplementary file 1 [file DataSheet1.docx]

**1 Supplementary data**

**1.1 Knowledge-based statements**

The statement *“in some circumstances, a woman applying for a job who does not give permission to access her medical record might lose the job opportunity”* is false (**Table S1**). The bivariate analysis portrayed women who have a college degree (OR 0.79, 95% CI 0.64-0.96, p=0.0205) or a high school degree or below (OR 0.74, 95% CI 0.55-1.00, p=0.0501) had lower odds of giving a wrong answer. On the other hand, women who were not married (OR 1.25, 95% CI 1.01-1.55, p=0.0435), who were retired (OR 1.45, 95% CI 1.18-1.78, p=0.0003) and who were in the 60-70-year-old bracket (OR 1.64, 1.37-1.95, p = 0.0001) had higher odds of giving a wrong answer. The multivariate analysis presented a similar pattern of associations except for the inclusion of the insurance variable which showed that women who did not have life insurance or were not interested in having one (OR 0.71, 95% CI 0.52-0.96, p=0.0250) had lower odds of giving a wrong answer.

The statement *“The level of breast cancer risk has no impact on a woman's ability to get a job”* is true (**Table S2**). The bivariate analysis revealed that women with a family history of breast cancer (OR 0.82, 95% CI 0.66-1.02, p=0.0782) and women from Quebec (OR 1.24, 95% CI 1.00-1.54, p=0.0508) had higher odds of giving the wrong answer. These results remained unchanged in multivariate analyses.

The statement “*employers may request access to a woman's medical record when she is applying for a job (including her level of breast cancer risk), to assess specific health aspects related to the job”* is false (**Table S3**)., those who did not have insurance or were not interested in having insurance (OR 0.62, 95% CI 0.44-0.87, p=0.0060), and those who had a high school degree or lower (OR 0.62, 95% CI 0.43-0.91, p=0.0132) had lower odds of giving the wrong answer. On the other hand, women from Quebec (OR 1.44, 95% CI 1.17-1.78, p=0.0007), women who had a family history of breast cancer (OR 1.42, 95% CI 1.15-1.75, p=0.0012) and who were aged 40-49 years (OR 1.70, 95% CI 1.27-2.27, p=0.0003) had higher odds of giving a wrong answer. A similar pattern of associations was observed in multivariate analyses except the fact that women who were retired (OR 1.35, 95% CI 1.00-1.82, p=0.0490) were found to have higher odds of giving a wrong answer.

**1.2 Perception-based statements**

The statement *“a woman's insurance might be cancelled if important health information (including level of breast cancer risk) is not given when buying or renewing life or health insurance”* aims to highlight the level of protection felt by the participants (**Table S4**). The bivariate analysis highlighted that women who were part of a visible minority (OR 0.50, 95% CI 0.30-0.81, p=0.0055), women who had a high school degree or less (OR 0.60, 95% CI 0.40-0.89, p=0.0110), and women who were unemployed (OR 0.36, 95% CI 0.23-0.56, p<0.0001) were less likely to feel unprotected. On the other hand, women who were not married/common law (OR 1.36, 95% CI 0.97-1.91, p=0.0786) had higher odds of feeling unprotected. The multivariate analysis presented a similar pattern of associations except for the exclusion of the married/common law variable.

The statement *“the level of breast cancer risk has no impact on a woman's ability to buy insurance”* aims to highlight the level of protection felt by the participants (**Table S5**). The bivariate analysis revealed that women from Quebec (OR 0.82, 95% CI 0.68-0.99, p=0.0368), women with higher-than-average risks of developing breast cancer (OR 0.74, 95% CI 0.58-0.95, p=0.0176), women with a college degree (OR 0.68, 95% CI 0.56-0.84, p=0.0003), a high school degree or lower (OR 0.59, 95% CI 0.44-0.79, p=0.0005), and women who are retired (OR 0.75, 95% CI 0.61-0.92, p=0.0064) or unemployed (OR 0.60, 95% CI 0.41-0.88, p=0.0082) had lower odds of feeling unprotected. The multivariate analysis demonstrated the same results.

**2 Supplementary Tables**

| **Table S1. Bivariate and Multivariate analysis considering sociodemographic characteristics for Question 1 on knowledge of the law.** | | | | | | | | | |
| --- | --- | --- | --- | --- | --- | --- | --- | --- | --- |
| **Sociodemographic factors** |  | **Bivariate analysis** | | | | **Multivariate analysis** | | | |
|  |  | **Doesn't know** |  | **Wrong answer** |  | **Doesn't know** |  | **Wrong answer** |  |
|  |  | **vs** |  | **vs** |  | **vs** |  | **vs** |  |
|  |  | **Correct answer** |  | **Correct answer** |  | **Correct answer** |  | **Correct answer** |  |
|  |  | **OR (95% CI)** | **p value** | **OR (95% CI)** | **p value** | **OR (95% CI)** | **p value** | **OR (95% CI)** | **p value** |
| **Study Site** |  |  |  |  |  |  |  |  |  |
|  | **QC** | 0.58 (0.49-0.68) | **<0.0001** | 0.97 (0.80-1.16) | 0.7095 | 0.66 (0.54-0.80) | **<0.0001** | 0.95 (0.76-1.18) | 0.6143 |
|  | **ON** | 1 |  | 1 |  |  |  |  |  |
| **Age** |  |  |  |  |  |  |  |  |  |
|  | **40 - 49** | 0.76 (0.59-0.99) | **0.0388** | 1.08 (0.82-1.41) | 0.5827 | 0.98 (0.73-1.31) | 0.8940 | 1.13 (0.84-1.53) | 0.4202 |
|  | **50 - 59** | 1 |  | 1 |  |  |  |  |  |
|  | **60 - 70** | 1.64 (1.37-1.95) | **<0.0001** | 1.27 (1.04-1.56) | **0.0212** | 1.51 (1.21-1.88) | **0.0003** | 1.14 (0.88-1.47) | 0.3309 |
| **Nativity** |  |  |  |  |  |  |  |  |  |
|  | **Born in Canada** | 1 |  | 1 |  |  |  |  |  |
|  | **Born outside of Canada** | 0.97 (0.76-1.24) | 0.8216 | 0.95 (0.71-1.26) | 0.7089 |  |  |  |  |
| **Visible minority** |  |  |  |  |  |  |  |  |  |
|  | **Not a visible minority** | 1 |  | 1 |  |  |  |  |  |
|  | **Visible minority** | 0.99 (0.70-1.39) | 0.9432 | 0.77 (0.50-1.17) | 0.2168 |  |  |  |  |
| **Risk level letter** |  |  |  |  |  |  |  |  |  |
|  | **Average risk** | 1 |  | 1 |  |  |  |  |  |
|  | **High risk** | 0.77 (0.52-1.13) | 0.1792 | 0.93 (0.61-1.41) | 0.7202 |  |  |  |  |
|  | **Higher than average risk** | 0.90 (0.72-1.13) | 0.3651 | 1.07 (0.84-1.37) | 0.5922 |  |  |  |  |
| **Family history of BC** |  |  |  |  |  |  |  |  |  |
|  | **No family history of BC** | 1 |  | 1 |  |  |  |  |  |
|  | **Family history of BC** | 0.89 (0.76-1.05) | 0.1586 | 1.11 (0.92-1.34) | 0.2709 | 0.99 (0.83-1.18) | 0.9275 | 1.18 (0.96-1.44) | 0.1088 |
| **Life or personal health insurance)** |  |  |  |  |  |  |  |  |  |
|  | **No/not interested in insurance** | 1.13 (0.89-1.43) | 0.3221 | 0.84 (0.63-1.12) | 0.2303 | 0.89 (0.70-1.15) | 0.3780 | 0.71 (0.52-0.96) | **0.0250** |
|  | **Has insurance** | 1 |  | 1 |  |  |  |  |  |
| **Marital status** |  |  |  |  |  |  |  |  |  |
|  | **Married** | 1 |  | 1 |  |  |  |  |  |
|  | **Single** | 1.04 (0.86-1.27) | 0.6724 | 1.25 (1.01-1.55) | **0.0435** | 1.00 (0.82-1.24) | 0.9759 | 1.31 (1.05-1.64) | **0.0183** |
| **Education** |  |  |  |  |  |  |  |  |  |
|  | **Highschool and below** | 0.88 (0.68-1.14) | 0.3217 | 0.74 (0.55-1.00) | **0.0501** | 0.82 (0.62-1.08) | 0.1627 | 0.73 (0.53-1.01) | 0.0572 |
|  | **College** | 0.88 (0.74-1.06) | 0.1745 | 0.79 (0.64-0.97) | **0.0205** | 0.91 (0.75-1.10) | 0.3124 | 0.80 (0.65-0.99) | **0.0433** |
|  | **Bachelor's and above** | 1 |  | 1 |  |  |  |  |  |
| **Employment** |  |  |  |  |  |  |  |  |  |
|  | **Employed** | 1 |  | 1 |  |  |  |  |  |
|  | **Retired** | 1.60 (1.34-1.92) | **<0.0001** | 1.45 (1.18-1.78) | **0.0003** | 1.26 (1.00-1.59) | **0.0506** | 1.51 (1.16-1.97) | **0.0023** |
|  | **Unemployed** | 1.26 (0.91-1.75) | 0.1668 | 1.06 (0.72-1.56) | 0.7714 | 1.19 (0.83-1.70) | 0.3417 | 1.11 (0.73-1.69) | 0.6224 |
| OR: Odd ratio; CI: Confidence interval; BC: Breast Cancer; p values <0.1 (significant bivariate results) and <0.05 (significant multivariate results) are in bold | | | | | | | | | |

| **Table S2. Bivariate and Multivariate analysis considering sociodemographic characteristics for Question 2 on knowledge of the law.** | | | | | | | | | |
| --- | --- | --- | --- | --- | --- | --- | --- | --- | --- |
| **Sociodemographic factors** |  | **Bivariate analysis** | | | | **Multivariate analysis** | | | |
|  |  | **Doesn't know** |  | **Wrong answer** |  | **Doesn't know** |  | **Wrong answer** |  |
|  |  | **vs** |  | **vs** |  | **vs** |  | **vs** |  |
|  |  | **Correct answer** |  | **Correct answer** |  | **Correct answer** |  | **Correct answer** |  |
|  |  | **OR (95% CI)** | **p value** | **OR (95% CI)** | **p value** | **OR (95% CI)** | **p value** | **OR (95% CI)** | **p value** |
| **Study Site** |  |  |  |  |  |  |  |  |  |
|  | **QC** | 0.79 (0.66-0.94) | **0.0086** | 1.24 (1.00-1.54) | **0.0508** | 0.83 (0.69-0.99) | **0.0382** | 1.27 (1.02-1.59) | **0.0352** |
|  | **ON** | 1 |  | 1 |  |  |  |  |  |
| **Age** |  |  |  |  |  |  |  |  |  |
|  | **40 - 49** | 0.91 (0.69-1.21) | 0.5265 | 1.02 (0.75-1.41) | 0.8876 |  |  |  |  |
|  | **50 - 59** | 1 |  | 1 |  |  |  |  |  |
|  | **60 - 70** | 1.38 (1.14-1.66) | **0.0009** | 0.95 (0.75-1.20) | 0.6552 |  |  |  |  |
| **Nativity** |  |  |  |  |  |  |  |  |  |
|  | **Born in Canada** | 1 |  | 1 |  |  |  |  |  |
|  | **Born outside of Canada** | 1.18 (0.91-1.53) | 0.2142 | 1.28 (0.94-1.75) | 0.1172 |  |  |  |  |
| **Visible minority** |  |  |  |  |  |  |  |  |  |
|  | **Not a visible minority** | 1 |  | 1 |  |  |  |  |  |
|  | **Visible minority** | 1.12 (0.78-1.62) | 0.5348 | 0.96 (0.60-1.55) | 0.8783 |  |  |  |  |
| **Risk level** |  |  |  |  |  |  |  |  |  |
|  | **Average risk** | 1 |  | 1 |  |  |  |  |  |
|  | **High risk** | 0.71 (0.46-1.09) | 0.1150 | 0.68 (0.40-1.17) | 0.1665 |  |  |  |  |
|  | **Higher than average risk** | 0.90 (0.71-1.13) | 0.3513 | 0.79 (0.58-1.07) | 0.1213 |  |  |  |  |
| **Family history of BC** |  |  |  |  |  |  |  |  |  |
|  | **No family history of BC** | 1 |  | 1 |  |  |  |  |  |
|  | **Family history of BC** | 0.88 (0.74-1.04) | 0.1343 | 0.82 (0.66-1.02) | **0.0782** | 0.92 (0.77-1.10) | 0.3396 | 0.78 (0.62-0.97) | **0.0246** |
| **Life or personal health insurance** |  |  |  |  |  |  |  |  |  |
|  | **No/not interested in insurance** | 1.04 (0.81-1.34) | 0.7655 | 0.80 (0.57-1.12) | 0.1849 |  |  |  |  |
|  | **Has insurance** | 1 |  | 1 |  |  |  |  |  |
| **Marital status** |  |  |  |  |  |  |  |  |  |
|  | **Married** | 1 |  | 1 |  |  |  |  |  |
|  | **Single** | 0.97 (0.79-1.20) | 0.7874 | 1.24 (0.97-1.58) | 0.0905 |  |  |  |  |
| **Education** |  |  |  |  |  |  |  |  |  |
|  | **Highschool and below** | 0.64 (0.48-0.87) | **0.0036** | 0.89 (0.63-1.25) | 0.5059 | 0.59 (0.44-0.80) | **0.0007** | 0.93 (0.65-1.31) | 0.6602 |
|  | **College** | 0.81 (0.67-0.98) | **0.0316** | 0.91 (0.72-1.15) | 0.4053 | 0.79 (0.65-0.96) | **0.0177** | 0.90 (0.71-1.14) | 0.3643 |
|  | **Bachelor's and above** | 1 |  | 1 |  |  |  |  |  |
| **Employment** |  |  |  |  |  |  |  |  |  |
|  | **Employed** | 1 |  | 1 |  |  |  |  |  |
|  | **Retired** | 1.37 (1.14-1.65) | **0.0009** | 0.87 (0.68-1.10) | 0.2453 | 1.39 (1.15-1.68) | **0.0008** | 0.90 (0.70-1.15) | 0.3908 |
|  | **Unemployed** | 0.99 (0.69-1.42) | 0.9399 | 0.73 (0.46-1.17) | 0.1909 | 1.01 (0.70-1.46) | 0.9389 | 0.73 (0.45-1.18) | 0.1938 |
| OR: Odd ratio; CI: Confidence interval; BC: Breast Cancer; p values <0.1 (significant bivariate results) and <0.05 (significant multivariate results) are in bold | | | | | | | | | |

| **Table S3. Bivariate and Multivariate analysis considering sociodemographic characteristics for Question 3 on knowledge of the law.** | | | | | | | | | | |
| --- | --- | --- | --- | --- | --- | --- | --- | --- | --- | --- |
| **Sociodemographic factors** |  | **Bivariate analysis** | | | | | **Multivariate analysis** | | | |
|  |  | | **Doesn't know** |  | **Wrong answer** |  | **Doesn't know** |  | **Wrong answer** |  |
|  |  | | **vs** |  | **vs** |  | **vs** |  | **vs** |  |
|  |  | | **Correct answer** |  | **Correct answer** |  | **Correct answer** |  | **Correct answer** |  |
|  |  | | **OR (95% CI)** | **p value** | **OR (95% CI)** | **p value** | **OR (95% CI)** | **p value** | **OR (95% CI)** | **p value** |
| **Study Site** |  | |  |  |  |  |  |  |  |  |
|  | **QC** | | 0.65 (0.55-0.77) | **<0.0001** | 1.44 (1.17-1.78) | **0.0007** | 0.68 (0.55-0.83) | **0.0002** | 1.21 (0.95-1.56) | 0.1272 |
|  | **ON** | | 1 |  | 1 |  |  |  |  |  |
| **Age** |  | |  |  |  |  |  |  |  |  |
|  | **40 - 49** | | 1.00 (0.76-1.32) | 0.9848 | 1.70 (1.27-2.27) | **0.0003** | 1.29 (0.95-1.76) | 0.1026 | 1.45 (1.05-2.00) | **0.0225** |
|  | **50 - 59** | | 1 |  | 1 |  |  |  |  |  |
|  | **60 - 70** | | 1.48 (1.24-1.78) | **<0.0001** | 1.03 (0.82-1.30) | 0.8026 | 1.42 (1.14-1.78) | **0.0021** | 1.00 (0.75-1.34) | 0.9904 |
| **Nativity** |  | |  |  |  |  |  |  |  |  |
|  | **Born in Canada** | | 1 |  | 1 |  |  |  |  |  |
|  | **Born outside of Canada** | | 0.77 (0.59-1.00) | **0.0463** | 0.78 (0.56-1.08) | 0.1300 |  |  |  |  |
| **Visible minority** |  | |  |  |  |  |  |  |  |  |
|  | **Not a visible minority** | | 1 |  | 1 |  |  |  |  |  |
|  | **Visible minority** | | 0.66 (0.45-0.97) | **0.0337** | 0.79 (0.50-1.26) | 0.3242 |  |  |  |  |
| **Risk level** |  | |  |  |  |  |  |  |  |  |
|  | **Average risk** | | 1 |  | 1 |  |  |  |  |  |
|  | **High risk** | | 1.12 (0.77-1.63) | 0.5634 | 1.04 (0.64-1.69) | 0.8691 |  |  |  |  |
|  | **Higher than average risk** | | 0.91 (0.72-1.14) | 0.3880 | 0.93 (0.70-1.23) | 0.5969 |  |  |  |  |
| **Family history of BC** |  | |  |  |  |  |  |  |  |  |
|  | **No family history of BC** | | 1 |  | 1 |  |  |  |  |  |
|  | **Family history of BC** | | 0.94 (0.80-1.11) | 0.4764 | 1.42 (1.15-1.75) | **0.0012** | 1.02 (0.86-1.23) | 0.7931 | 1.25 (1.00-1.56) | **0.0485** |
| **Life or personal health insurance** |  | |  |  |  |  |  |  |  |  |
|  | **No/not interested in insurance** | | 1.02 (0.80-1.29) | 0.9033 | 0.62 (0.44-0.87) | **0.0060** | 0.84 (0.66-1.09) | 0.1847 | 0.65 (0.45-0.93) | **0.0190** |
|  | **Has insurance** | | 1 |  | 1 |  |  |  |  |  |
| **Marital status** |  | |  |  |  |  |  |  |  |  |
|  | **Married** | | 1 |  | 1 |  |  |  |  |  |
|  | **Single** | | 1.13 (0.93-1.37) | 0.2281 | 1.11 (0.87-1.41) | 0.4007 |  |  |  |  |
| **Education** |  | |  |  |  |  |  |  |  |  |
|  | **Highschool and below** | | 1.08 (0.84-1.41) | 0.5442 | 0.62 (0.43-0.91) | **0.0132** | 1.02 (0.77-1.35) | 0.9040 | 0.65 (0.44-0.97) | **0.0349** |
|  | **College** | | 1.02 (0.85-1.23) | 0.8016 | 0.93 (0.74-1.16) | 0.5076 | 1.06 (0.87-1.28) | 0.5903 | 0.94 (0.74-1.19) | 0.5967 |
|  | **Bachelor's and above** | | 1 |  | 1 |  |  |  |  |  |
| **Employment** |  | |  |  |  |  |  |  |  |  |
|  | **Employed** | | 1 |  | 1 |  |  |  |  |  |
|  | **Retired** | | 1.37 (1.14-1.64) | **0.0006** | 1.03 (0.82-1.30) | 0.7969 | 1.15 (0.91-1.44) | 0.2398 | 1.35 (1.00-1.82) | **0.0490** |
|  | **Unemployed** | | 1.55 (1.12-2.14) | **0.0087** | 1.01 (0.65-1.58) | 0.9516 | 1.36 (0.95-1.95) | 0.0929 | 1.34 (0.85-2.12) | 0.1272 |
| OR: Odd ratio; CI: Confidence interval; BC: Breast Cancer; p values <0.1 (significant bivariate results) and <0.05 (significant multivariate results) are in bold | | | | | | | | | | |

| **Table S4. Bivariate and Multivariate analysis considering sociodemographic characteristics for Question 4 on knowledge of the law.** | | | | | | | | | | |
| --- | --- | --- | --- | --- | --- | --- | --- | --- | --- | --- |
| **Sociodemographic factors** |  | **Bivariate analysis** | | | | **Multivariate analysis** | | | | |
|  |  | **Doesn't feel protected** |  | **Doesn't know** |  | **Doesn't feel protected** |  | | **Doesn't know** |  |
|  |  | **vs** |  | **vs** |  | **vs** | |  | **vs** |  |
|  |  | **Feels protected** |  | **Feels protected** |  | **Feels protected** |  | | **Feels protected** |  |
|  |  | **OR (95% CI)** | **p value** | **OR (95% CI)** | **p value** | **OR (95% CI)** | **p value** | | **OR (95% CI)** | **p value** |
| **Study Site** |  |  |  |  |  |  |  | |  |  |
|  | **QC** | 1.25 (0.95-1.63) | 0.1108 | 0.65 (0.49-0.86) | **0.0028** | 1.20 (0.87-1.66) | 0.2618 | | 0.69 (0.49-0.97) | **0.0311** |
|  | **ON** | 1 |  | 1 |  |  |  | |  |  |
| **Age** |  |  |  |  |  |  |  | |  |  |
|  | **40 - 49** | 1.05 (0.71-1.56) | 0.8062 | 0.72 (0.47-1.12) | 0.137 | 0.86 (0.55-1.34) | 0.5093 | | 0.82 (0.51-1.33) | 0.421 |
|  | **50 - 59** | 1 |  | 1 |  |  |  | |  |  |
|  | **60 - 70** | 1.07 (0.80-1.44) | 0.6597 | 1.42 (1.05-1.94) | **0.0249** | 1.20 (0.84-1.72) | 0.3180 | | 1.51 (1.04-2.19) | **0.0294** |
| **Nativity** |  |  |  |  |  |  |  | |  |  |
|  | **Born in Canada** | 1 |  | 1 |  |  |  | |  |  |
|  | **Born outside of Canada** | 0.74 (0.50-1.10) | 0.1408 | 1.11 (0.74-1.67) | 0.6191 |  |  | |  |  |
| **Visible minority** |  |  |  |  |  |  |  | |  |  |
|  | **Not a visible minority** | 1 |  | 1 |  |  |  | |  |  |
|  | **Visible minority** | 0.49 (0.30-0.81) | **0.0055** | 0.80 (0.48-1.32) | 0.3796 | 0.49 (0.29-0.84) | **0.0086** | | 0.66 (0.39-1.13) | 0.1291 |
| **Risk level** |  |  |  |  |  |  |  | |  |  |
|  | **Average risk** | 1 |  | 1 |  |  |  | |  |  |
|  | **High risk** | 1.18 (0.59-2.40) | 0.6349 | 1.59 (0.77-3.27) | 0.2069 |  |  | |  |  |
|  | **Higher than average risk** | 0.99 (0.69-1.43) | 0.9581 | 1.08 (0.74-1.58) | 0.6922 |  |  | |  |  |
| **Family history of BC** |  |  |  |  |  |  |  | |  |  |
|  | **No family history of BC** | 1 |  | 1 |  |  |  | |  |  |
|  | **Family history of BC** | 1.00 (0.76-1.30) | 0.9698 | 0.84 (0.63-1.11) | 0.2209 | 0.96 (0.73-1.27) | 0.7737 | | 0.89 (0.67-1.20) | 0.4551 |
| **Life or personal health insurance** |  |  |  |  |  |  |  | |  |  |
|  | **No/not interested in insurance** | 0.79 (0.53-1.16) | 0.2249 | 1.10 (0.74-1.65) | 0.6290 |  |  | |  |  |
|  | **Has insurance** | 1 |  | 1 |  |  |  | |  |  |
| **Marital status** |  |  |  |  |  |  |  | |  |  |
|  | **Married** | 1 |  | 1 |  |  |  | |  |  |
|  | **Single** | 1.36 (0.97-1.91) | **0.0786** | 1.26 (0.88-1.79) | 0.2067 |  |  | |  |  |
| **Education** |  |  |  |  |  |  |  | |  |  |
|  | **Highschool and below** | 0.60 (0.40-0.89) | **0.0110** | 0.77 (0.51-1.17) | 0.2263 | 0.62 (0.41-0.95) | **0.0265** | | 0.72 (0.47-1.12) | 0.1489 |
|  | **College** | 0.80 (0.59-1.08) | 0.1353 | 0.94 (0.69-1.29) | 0.7034 | 0.78 (0.58-1.06) | 0.1141 | | 0.96 (0.70-1.32) | 0.8084 |
|  | **Bachelor's and above** | 1 |  | 1 |  |  |  | |  |  |
| **Employment** |  |  |  |  |  |  |  | |  |  |
|  | **Employed** | 1 |  | 1 |  |  |  | |  |  |
|  | **Retired** | 0.85 (0.63-1.15) | 0.2946 | 1.06 (0.78-1.45) | 0.7127 | 0.79 (0.54-1.15) | 0.2124 | | 0.78 (0.53-1.16) | 0.2217 |
|  | **Unemployed** | 0.36 (0.23-0.56) | **<0.0001** | 0.51 (0.32-0.80) | **0.0035** | 0.37 (0.24-0.58) | **<0.0001** | | 0.47 (0.29-0.75) | **0.0014** |
| OR: Odd ratio; CI: Confidence interval; BC: Breast Cancer; p values <0.1 (significant bivariate results) and <0.05 (significant multivariate results) are in bold | | | | | | | | | | |

| **Table S5. Bivariate and Multivariate analysis considering sociodemographic characteristics for Question 5 on knowledge of the law.** | | | | | | | | | | |
| --- | --- | --- | --- | --- | --- | --- | --- | --- | --- | --- |
| **Sociodemographic factors** |  | **Bivariate analysis** | | | | **Multivariate analysis** | | | | |
|  |  | **Doesn't feel protected** |  | **Doesn't know** |  | **Doesn't feel protected** | |  | **Doesn't know** |  |
|  |  | **vs** |  | **vs** |  | **vs** | |  | **vs** |  |
|  |  | **Feels protected** |  | **Feels protected** |  | **Feels protected** | |  | **Feels protected** |  |
|  |  | **OR (95% CI)** | **p value** | **OR (95% CI)** | **p value** | **OR (95% CI)** | | **p value** | **OR (95% CI)** | **p value** |
| **Study Site** |  |  |  |  |  | |  |  |  |  |
|  | **QC** | 0.82 (0.68-0.99) | **0.0368** | 0.57 (0.47-0.71) | **<0.0001** | 0.77 (0.63-0.94) | | **0.0101** | 0.57 (0.46-0.71) | **<0.0001** |
|  | **ON** | 1 |  | 1 |  |  | |  |  |  |
| **Age** |  |  |  |  |  |  | |  |  |  |
|  | **40 - 49** | 1.00 (0.76-1.32) | 0.9920 | 0.70 (0.50-0.97) | **0.0311** |  | |  |  |  |
|  | **50 - 59** | 1 |  | 1 |  |  | |  |  |  |
|  | **60 - 70** | 0.86 (0.70-1.06) | 0.1563 | 1.26 (1.01-1.57) | **0.0423** |  | |  |  |  |
| **Nativity** |  |  |  |  |  |  | |  |  |  |
|  | **Born in Canada** | 1 |  | 1 |  |  | |  |  |  |
|  | **Born outside of Canada** | 0.97 (0.73-1.31) | 0.8609 | 1.30 (0.96-1.77) | **0.0921** |  | |  |  |  |
| **Visible minority** |  |  |  |  |  |  | |  |  |  |
|  | **Not a visible minority** | 1 |  | 1 |  |  | |  |  |  |
|  | **Visible minority** | 0.93 (0.62-1.38) | 0.7053 | 0.99 (0.64-1.52) | 0.9551 |  | |  |  |  |
| **Risk level** |  |  |  |  |  |  | |  |  |  |
|  | **Average risk** | 1 |  | 1 |  |  | |  |  |  |
|  | **High risk** | 1.01 (0.65-1.57) | 0.9609 | 1.01 (0.63-1.64) | 0.9543 | 0.99 (0.63-1.58) | | 0.9810 | 1.24 (0.75-2.06) | 0.3976 |
|  | **Higher than average risk** | 0.74 (0.58-0.95) | **0.0176** | 0.84 (0.65-1.10) | 0.1984 | 0.73 (0.57-0.95) | | **0.0171** | 0.89 (0.68-1.17) | 0.4049 |
| **Family history of BC** |  |  |  |  |  |  | |  |  |  |
|  | **No family history of BC** | 1 |  | 1 |  |  | |  |  |  |
|  | **Family history of BC** | 0.95 (0.78-1.14) | 0.5673 | 0.87 (0.71-1.06) | 0.1716 | 1.02 (0.83-1.24) | | 0.8681 | 0.96 (0.77-1.19) | 0.6987 |
| **Life or personal health insurance** |  |  |  |  |  |  | |  |  |  |
|  | **No/not interested in insurance** | 1.04 (0.78-1.39) | 0.7939 | 1.50 (1.11-2.03) | **0.0082** |  | |  |  |  |
|  | **Has insurance** | 1 |  | 1 |  |  | |  |  |  |
| **Marital status** |  |  |  |  |  |  | |  |  |  |
|  | **Married** | 1 |  | 1 |  |  | |  |  |  |
|  | **Single** | 1.22 (0.97-1.53) | 0.0834 | 1.18 (0.93-1.51) | 0.1761 |  | |  |  |  |
| **Education** |  |  |  |  |  |  | |  |  |  |
|  | **Highschool and below** | 0.59 (0.44-0.79) | **0.0005** | 0.84 (0.61-1.14) | 0.2604 | 0.64 (0.47-0.86) | | **0.0032** | 0.81 (0.59-1.11) | 0.1818 |
|  | **College** | 0.68 (0.56-0.84) | **0.0003** | 0.77 (0.62-0.96) | **0.021** | 0.71 (0.58-0.87) | | **0.0011** | 0.78 (0.63-0.98) | **0.0349** |
|  | **Bachelor's and above** | 1 |  | 1 |  |  | |  |  |  |
| **Employment** |  |  |  |  |  |  | |  |  |  |
|  | **Employed** | 1 |  | 1 |  |  | |  |  |  |
|  | **Retired** | 0.75 (0.61-0.92) | **0.0064** | 1.19 (0.96-1.48) | 0.1180 | 0.75 (0.61-0.93) | | **0.0079** | 1.13 (0.90-1.41) | 0.305 |
|  | **Unemployed** | 0.60 (0.41-0.88) | **0.0082** | 1.04 (0.71-1.54) | 0.8273 | 0.63 (0.43-0.92) | | **0.0165** | 1.00 (0.67-1.49) | 0.9972 |
| OR: Odd ratio; CI: Confidence interval; BC: Breast Cancer; p values <0.1 (significant bivariate results) and <0.05 (significant multivariate results) are in bold | | | | | | | | | | |
